# Brazilian scientific journals: challenges, (dis)incentives and one fundamental question

**DOI:** 10.1590/0074-02760170001

**Published:** 2017-10

**Authors:** Adeilton Brandão, Elisa Cupolillo, Claude Pirmez

There are more than a thousand journals that publish research results from all knowledge fields in Brazil. Despite the existence of centennial journals such as “*Memorias do Instituto Oswaldo Cruz*” and “*Anais da Academia Brasileira de Ciencias*”, which started publishing in 1907 and 1929, respectively, Brazilian researchers do not consider international journals published here as a source of academic prestige. Following the steady growth of Brazilian science in the last quarter of the 20th century, Brazilian researchers have been engaged in an evaluative and rewarding system that has pushed them to seek out journals deemed as offering more influence and visibility in the academic world. These journals are largely owned by major publishers based in the USA or Europe.

We believe that it is time to examine reasons for this misalignment between rapid scientific output over the last 40 years in Brazil and the loss (or absence) of prestige of Brazilian journals. We present at least five key points for reflection:

1. Why does the Brazilian scientific environment not offer incentives for researchers to submit the best scientific findings to Brazilian journals despite the availability of dozens of journals in each major field?
2. Can Brazilian journals be considered good vehicles of knowledge dissemination according to good publishing practices?
3. Should we identify a given number of journals to be supported as “world class journals” through our major funding agencies?
4. What influence should funding agencies (CNPq, CAPES, and FAPs) have over this process? Should they develop a specific strategy for scientific publishing?
5. What are the roles of the science education and graduate programmes, universities, and scholar societies, e.g., the Brazilian Academy of Sciences and the Brazilian Association for Science Advancement?

Answering these questions will involve rigorous data collection and the analysis of all stakeholders involved in the publication of science in Brazil, which is not feasible over the very short term. However, some thoughts are worth noting.

Under the existing national Science & Technology system, journals published in Brazil in the natural sciences (biology, physics and chemistry) face major barriers in terms of attracting manuscripts reporting the best results from Brazilian researchers. Among these reasons, one is most prominent: researchers are not incentivised to publish articles in Brazilian journals, rendering these journals the last choice for manuscript submission. These incentives are grounded in two factors: (a) a strong emphasis on the journal impact factor, an index that ranks Brazilian journals last; (b) an understanding that manuscript analysis from non-Brazilian editors and peers imprints prestige, facilitates grant approval and (especially for younger researchers) boosts career prospects.

The case of science publishing in Brazil shows that we are in urgent need of a virtuous publishing cycle: *a journal that publishes high quality research motivates researchers to send new manuscripts of similar quality to those already published, thus contributing to an increase in journal prestige and rank, which in turn leads to the submission of more strong manuscripts and so on*. In fact, we have a vicious publishing cycle: *journals publishing less novel research that does not motivate researchers to submit their best manuscripts to these journals, which in turn continue to receive and publish less novel research results, compromising the rank and prestige of such journals.*

Our journals thus face the challenge of ending this vicious cycle. Rules set out by Brazilian graduate education agencies have to a large extent made it difficult for journals to exit this vicious cycle through the use of their own resources and practices. One strategy could involve the promotion of an innovative culture that can shift the focus from “where research has been published” to “what has been published”. An additional pathway may involve the integration of journals into science internationalization efforts in Brazil. However, what does science internationalisation mean? If we ask a typical researcher, he/she might give us the following two definitions: (i) collaborating with foreign researchers and publishing exclusively in non-Brazilian journals; (ii) collaborating or not collaborating with foreign researchers and publishing in Brazilian journals that meet requirements for science publishing at the global level.

From an editorial perspective, any Brazilian journal aspiring to become “international” must exhibit the following attributes: the use of English as a *lingua franca*, professionalism, the employment of academic and/or full-time editors, editorial innovation, sustainability, credibility, adherence to good publishing practices, the use of adequate technology infrastructures, open access capabilities, and editorial transparency.

Regardless of which option best suits our academic culture and national regulations, we must address a fundamental question: “Should researchers and funding agencies support the editing of ***world class journals*** in Brazil?” In other words, “Do we really wish to nurture in this country journals with published articles that boost the prestige of authors?”.

Let us spread the word to scientists and planners of the Brazilian national strategy for science, technology and innovation!

*Adeilton Brandão* *Elisa Cupolillo* *Claude Pirmez* Editors

